# The psychometric properties and gender invariance of the Italian version of the Perceived Vulnerability to Disease Questionnaire (I-PVDQ) during the COVID-19 pandemic

**DOI:** 10.1186/s40359-022-01023-z

**Published:** 2022-12-29

**Authors:** Francesca Chiesi, Georgia Marunic, Carlotta Tagliaferro, Chloe Lau

**Affiliations:** 1 Department of Neuroscience, Psychology, Drug, and Child’s Health (NEUROFARBA), Section of Psychology, University of Florence, Via San Salvi 12-Padiglione 26, 50135 Florence, Italy; 2 School of Psychology, University of Florence, Florence, Italy; 3 Centre for Addiction and Mental Health, Toronto, ON Canada

**Keywords:** Psychological assessment, Perceived vulnerability, COVID-19

## Abstract

**Background:**

The Perceived Vulnerability to Disease Questionnaire (PVDQ) measures beliefs associated with personal susceptibility to infectious diseases and behaviors or perceptions in the presence of potential risk of pathogen transmission. Given the onset of the Severe Acute Respiratory Syndrome Coronavirus 2 global pandemic, otherwise known as the COVID-19 pandemic, the construct being measured may function differently based on affective, behavioral, and cognitive changes along with the need to change norms and lifestyles in a global context. The present study aims to test the psychometric properties and the gender invariance of the Italian adaptation of the PVDQ to confirm that the scale can be used with Italian-speaking people, and that it functions effectively during a pandemic.

**Methods:**

A total of 509 participants filled out an online questionnaire including the Italian version of the I-PVDQ (I-PVDQ) and several measures of psychological constructs. Reliability and factor analyses (single and multigroup) were conducted. Bayesian correlation tests and Bayesian independent sample *t*-tests were used to assess the validity of I-PVDQ.

**Results:**

Exploratory factor analysis supported the two-factor structure of the I-PVDQ, and factor loadings loaded appropriately onto perceived infectability (PI) and germ aversion (GA). In terms of invariance, the scale showed configural, metric, scalar, and strict invariance across genders. Decisive evidence in favor of correlation with the measure of COVID-19 related fears for both PI and GA was found. There was strong evidence for observed correlations with COVID-19 related constructs such as intolerance to uncertainty, psychological inflexibility, resilience, stress, and anxiety. Women showed higher GA than men, while there were no gender differences in PI.

**Conclusions:**

Taken together, these results suggest that the I-PVDQ confirms the psychometric properties of the original version and that can be used to detect PVD when it is affected by environmental circumstances since its functioning is preserved during a pandemic.

## Background

During the course of human evolution, humans were forced to face numerous pathogens, and their immune systems subsequently developed and adapted in response to them [1]. In addition to these innate and adaptive immune responses, individuals have also developed a proactive system that enables them to recognize and avoid potential risks that augment the existing reactive system [2]. Its functioning was explained by the evolutionary disease avoidance mechanism proposed by Faulkner et al. [3], later known as the Behavioural Immune System (BIS) [4]. The authors describe a set of proactive protective behaviors against infection as a complementary mechanism to the immune system. The execution of these behaviors linked to how vulnerable a person feels. Indeed, individual differences in perceived vulnerability activate to different extents the processes directed to avoid diseases, such as detecting cues that characterize ill subjects, to knowing and acting on the strategies that prevent the transmission [4–6].

Nonetheless, along with these individual differences, there are specific situations in which this disposition becomes pervasive. When the World Health Organisation declared the Severe Acute Respiratory Syndrome Coronavirus 2 (SARS-CoV-2) global pandemic, otherwise known as the COVID-19 pandemic, humankind was confronted with a new and potentially fatal disease. To contain the spread of the pandemic, most countries have imposed strong political and societal measures (e.g., use of facemasks, strict social distancing, washing hands frequently) introducing vast behavioral changes in individuals’ daily routine. Therefore, the COVID-19 pandemic provides a unique research framework to investigate peoples’ perceived vulnerability to disease (PVD).

To conduct this investigation, the problem is approached from a psychometric point of view. Before the pandemic, one of the most widely used scales to measure PVD was the Perceived Vulnerability to Disease Questionnaire (PVDQ) [7]. Based on Faulkner et al.’s [3] disease avoidance mechanisms studies, the scale measures beliefs associated with personal susceptibility to infectious diseases and behaviors or perceptions in the presence of potential risk of pathogen transmission. During the last two years, many papers have been published containing data collected after the pandemic outbreak administering the PVDQ (e.g., [8–15]). However, it is unclear if the questionnaire’s psychometric properties remain unchanged once the fear of infection contributes to the perception of a largely uncontrollable and unpredictable threat, and the necessity to avoid exposure to pathogens is highly stressed or even imposed by the government (e.g., the explicit requirement to avoid shaking hands and maintain a physical distance, to stay with face masks at the workplace, on public transport, etc.). At the onset of COVID-19, the disease etiology and course were uncertain, with no proven treatment protocols, no immunity, and no vaccines. Thus, the exponential spread and high mortality associated with the prevention measures proposed by health authorities added to the altered perception of a potential threat [16, 17]. In this setting, it becomes important to investigate whether the psychometric properties can be considered equivalent across times marked or not marked by a pandemic. Indeed, when the construct being measured becomes dramatically relevant and pervasive, such as the subjective perception of the exposure to COVID-19, the PVDQ may need to be adjusted in response to environmental changes [14, 18] because the measured construct is changing or is changed. Studies on the effects of COVID-19 have shown a negative impact on people's mental health [19, 20]. Concerns about the possibility of being infected, or for life itself, increased during the pandemic [21] and had a psychological impact (e.g., higher levels of anxiety, depression, and stress) [22, 23]. Moreover, the COVID-19 pandemic social distancing norms and intensive hygiene practices have changed the perception of the vulnerability to the desease. Therefore, researchers that have employed the PVDQ during the pandemic suggested that such variations may have altered the psychometric properties of the whole questionnaire and the single items. Do Bú and colleagues [24] find high response scores to some items because most respondents perceived themselves as highly susceptible to infections and disgusted with sharing items perceived as “dirty” (e.g., handling money, sharing the same water bottle). Thus, they have advanced doubts as to how this situation might have affected people's responses to some items of the scale. Indeed, when administered during a pandemic, items exploring disgust and perceived infectability may not produce the same variability of responses, and to capture that issues it is necessary to modify the scale. Therefore, using a scale created for situations not marked by the pandemic might result in biased measures of PVD until the psychometric properties of that scale are confirmed when a pandemic is on.

## Objectives

Starting from this premise, the present study has three objectives as described below.

*Factorial structure and reliability of the I-PVDQ (Aim 1)*. We aimed at conducting a psychometric analysis to confirm the factor structure and reliability of the Italian version of the VDQ (I-PVDQ) when PVD becomes pervasive as it happens during a pandemic. Specifically, two moderately correlated factors were proposed for the scale [7]: the first one was named Perceived Infectability (PI) which assesses personal beliefs about perceived vulnerability to illness (i.e., how easily a person believes he or she can be infected if exposed to a possible source of illness), the second one was called Germ Aversion (GA) and it measures the discomfort experienced when a potential pathogen transmission is more likely to occur. Both factors showed a satisfactory internal consistency, with a higher value for the PI when compared to GA. More recently, using the Spanish version of the PVDQ, Diaz and colleagues [25] supported the presence of these two factors (even if they proposed to analyze separately each dimension), and they reported that internal consistency was good for PI, but failed to reach an adequate value for GA. Similarly, Ferreira and colleagues [26] were able to replicate, the two-factor structure of the scale with a Portuguese sample, but good internal consistency was obtained only after eliminating five items from the questionnaire. By and large, only a few studies have reported satisfactory internal consistency for both subscales [7, 27, 28], while inadequate levels of internal consistency have often been reported for the GA subscale [7, 25, 26, 29–31] raising doubts about the appropriateness of the items that comprise it. For this shortcoming, different scoring solutions were proposed: (two subscales separate scores [27, 28, 30, 31, 35], overall score [3, 29, 31–33], and only PI subscale score [34]) and to reinforce the factorial structure and general psychometric properties of the questionnaire, frequently adopted solutions involved dropping off some of the original items [24–26, 35]. Thus, the first aim of the current study was to ascertain if, mantaining all the items of the original version, the I-PVDQ can be deemed bidimensional with adequate reliability of each factor.

*Invariance of the I-PVDQ across genders (Aim 2)*. The current study also aimed to test the invariance of the I-PVDQ across male and female respondents because gender is a relevant factor in most of the analyses concerning PVD but, to make unbiased between-group comparisons, it is necessary to provide evidence of measurement equivalence of the scale across groups [36]. Specifically, previous studies found differences in PVD between men and women, with women showing a higher level of Perceived Infectability [3, 7, 25, 37], and Germ Aversion [3, 7, 37]. These gender differences have been explained by the higher pathogen disgust sensitivity of women (e.g., [38, 39]) which increases their avoidance behaviors associated with a possible risk of infection through contact or physical proximity to other people. It has been suggested that the two processes underlying BIS are disgust emotion and the fear of contamination, which together activate our pathogen avoidance system [5, 6]. Such a mechanism is easily understood from an evolutionary point of view: the ability to identify likely sources of infection would allow people to avoid them and ensure a longer life expectancy. The higher levels of PI and GA reported for women reaffirm this very function of the BIS. Indeed, women's evolutionary role which sees them involved in identifying better possible fitness in partners to ensure healthy offspring and guarantee their survival has been widely adopted in the literature [40–42]. Thus, to confirm the presence of gender differences, it is necessary to test the I-PVDQ invariance and ascertain that the same scoring and interpretation rules can be used in subgroups of women and men. To the best of our knowledge, only one study has tested and confirmed measurement invariance across genders [24]. Indeed, Diaz et al. [25] showed that PI and GA have the same factorial structure across genders, but they did not perform an invariance analysis.

*Correlations with related constructs and gender differences (Aim 3)*. The study aimed to explore the pattern of relationships between the I-PVDQ dimensions and psychological constructs linked to the pandemic, such as fear of COVID-19 [43], psychological inflexibility [44–46], intolerance to uncertainty [47, 48], resiliency [49–51], well-being [52, 53], and psychological distress (anxiety, stress, and depression) [54, 55].

Validation studies of the PVDQ scale, for the most part, have assessed the construct in relation to measures of disgust sensitivity, hypochondria, health beliefs, and personality traits [7, 24–26]. However, Duncan and colleagues [7] argue that although there is a relationship between PVD, disgust sensitivity, and health beliefs, they are to be considered different constructs. Disgust sensitivity has to do with the triggering circumstances, health beliefs refer to general concerns and attitudes about the possibility of getting sick, while PVDQ specifically assesses beliefs about susceptibility to infectious diseases, intended as the extent to which people consider themselves vulnerable once exposed to certain pathogens, and the emotional distress this perception of risk causes [7, 56]. The COVID-19 pandemic affected people’s perceived vulnerability and increased fear of contamination levels. Therefore, PVD was placed in close relationship with several psychological constructs studied during the COVID-19 pandemic that allows studying the construct beyond the previously proposed framework. Fear is an adaptive emotion that enables coping with a potential threat like triggering safety behaviors (e.g., washing hands regularly) [57]. However, excessive or low levels of fear compared to the actual threat become maladaptive [58]. People who underestimate the risk may disregard government measures to slow the propagation of coronavirus and spread the disease, conversely excessive fear may lead to exaggerated safety behaviors that have been linked to mental health problems such as phobia, stress, or social anxiety [59–65]. High levels of fear related to infectious diseases such as COVID-19 can induce people not to reason rationally [66]. Several studies have been concerned with assessing the levels, causes, and consequences of Covid-19 pandemic-related fear [21, 59, 67–74]. If fear of COVID-19 altered safety behaviors, it should be possible to find this relationship between the two dimensions of the PVDQ and the Fear of COVID-19 Scale.

Another construct related to COVID-19 and PVD is the intolerance of uncertainty. When lacking sufficient information, a distorted cognitive appraisal can lead to the “fear of the unknown”. Intolerance of uncertainty is an individual disposition related to this ability [75] and is recognized as a cognitive vulnerability [76, 77] that can amplify the effects of stressors on mental health [78, 79]. Intolerance to uncertainty is considered a major vulnerability factor involved in the development of several psychological disorders [80]. Similarly, increased levels of stress, depression, and anxiety evidence the impact of COVID-19 on mental health outcomes [22, 61, 81–83]. Therefore, it is relevant to assess levels of depression, anxiety, and stress, along with intolerance to uncertainty in relation to PVD. In contrast, psychological resilience and psychological flexibility can have buffering effects when facing adversities and improve levels of psychological well-being. A large body of research supports the association between psychological flexibility and mental well-being. The moderating effect of psychological flexibility on stressor outcomes and negative life events has been proven [84, 85]. Conversely, psychological inflexibility negatively affects levels of depression and anxiety [86–88]. Similarly, high levels of resiliency, conceived as a result of the interactions between internal resilience factors and environmental factors [89], facilitate successful adaptation and promote positive changes when confronting adversities, trauma, and stress [90], and consequently provide mental health protection [91, 92]. Therefore, higher levels of psychological resilience and flexibility can provide protection from psychological disorders and increase levels of well-being. Studies employing the PVDQ have shown that despite the protective role of BIS, it can contribute to aversive responses toward people perceived as potentially infectious [2, 93]. Moreover, perceived vulnerability to disease induces people to engage in more proactive preventive behaviors (particularly beneficial behavior when the risk of contagion is high). As noted for protective behaviors, higher levels of PVD have also been associated with greater psychological distress, and increased levels of anxiety and depression [94, 95]. In line with the literature, it was expected to find a relationship between PVD and these variables during the pandemic. If this is the case, this study could provide evidence of a decisive impact of the pandemic on the subjective perception of infectability and the related behavioral responses, suggesting that given the highly stressful pandemic scenario (i.e., the specific prevention regulations implemented during the global COVID-19 pandemic that led to increased awareness of possible pathogen transmission sources), environmental variables affect not only PVD fluctuations [14, 94], but also the PVDQ properties.

Finally, gender differences were explored to highlight similarities and differences with the previous study [3, 7, 25, 37].

## Methods

### Participants and procedure

A total of 509 Italian participants (58.7% female, age range from 18 to 76 years, *M*_*age*_ = 27.24, *SD* = 12.16) were recruited. Sampling was based on the “snowball” method [96], in which undergraduate students from a large university in Central Italy were invited to participate in an online questionnaire study and were also encouraged to recruit their acquaintances and relatives to participate. Since, the minimum sample size is 200 for factor analyses with ordinal data [97, 98] (see below Statistical Analysis), the sample size was deemed adequate.

Data collection began in mid-March 2021 and was completed at the end of April 2021. At that time, Italy was going through a new (third) pandemic wave. The prevention measures adopted through ministerial decrees provided for the existence of geographical areas of high, medium, and low risk based on the incidence of COVID-19 cases recorded in proportion to the number of inhabitants. The areas of central Italy where data collection was concentrated fell within the high-risk zone. Throughout the country, there was a requirement to wear FFP2-type masks outdoors and in all indoor places, an interpersonal distancing of at least one meter, and a general curfew from 10 PM to 5 AM. In high-risk areas, all movements had to be justified by proven work requirements, situations of absolute necessity or health, preschool educational services were suspended, and educational activities in schools of all levels could be conducted only remotely (online). In addition, all recreational and entertainment activities were suspended, while restaurants, bars, and other facilities closed. Thus, during the period of data collection for this study, the normal course of daily life was severely hampered.

All participants provided informed consent and they voluntarily took part in the study. No compensation or incentives were provided. None of the tests included were intended to be clinical diagnostic, which would require direct administration by people with specific professional qualifications, but they are tests that measure individual dispositions that are generally used for research purposes. The questionnaire was built so that it could be self-administered using the written information provided as a foreword to the study and the specific completion instructions included as a foreword to each scale. Once completed, the data were automatically recorded, and no further action was required from participants.

The study was approved by the university’s local institutional review board (Commissione Etica per la Ricerca dell’Università degli Studi di Firenze, n. 148 - prot. 0134386).

### Measures

The online questionnaire consisted of the following scales.

*Italian Perceived Vulnerability to Disease Questionnaire* (I-PVDQ). The PVDQ [7] is a self-report measure that contains 15 questions investigating two distinct dimensions of Perceived Infectability (PI) with seven items (e.g., “If an illness is “going around”, I will get it.”) and Germ Aversion (GA) with eight items (e.g., “It really bothers me when people sneeze without covering their mouths.”). The responses are coded on a seven-point scale (with endpoints labeled “*strongly disagree*” and “*strongly agree*”). The Italian version was obtained following a standard translation procedure [99] by two Italian psychologists fluent in English who discussed the differences between the two translated versions. The wording of item 15 has been changed to make it more up-to-date and modified as follows: “I avoid using *public telephones* because of the risk that I may catch something from the previous user.” to “I avoid using *other people’s mobile phones or going to Internet point* because of the risk that I may catch something from the previous user” (Internet Point is a common way of defining the different types of public spaces, widely used in Italy, where computers and the Internet access are available. Public telephones have fallen into disuse, but Internet Points are the places that come closest to what used to be the sharing and use of public telephone booths.). Once a single version was obtained, a native English speaker fluent in Italian, who was not exposed to content from the original scale, back-translated it into Italian. The back-translation was largely similar to the original scale. Therefore, this preliminary version was presented to a small group of native Italians (*N* = 6) who were asked to read the items and judge whether they were understandable or to indicate any unclear/ambiguous word, sentence, or meaning. No problems were reported and thus, no further adjustments were made.

*Multidimensional Assessment of COVID-19-Related Fears* (MAC-RF) [43]. It is an eight-item self-report measure that assesses clinically relevant domains of fear (i.e., cognitive, relational, bodily, and behavioural) during the COVID-19 pandemic. Responses are collected on a 5-point Likert scale (ranging from 0 = *very unlike me* to *4* = *very like me*). A total score is obtained by summing up the eight-item answers. The higher it is, the stronger fears are. Cronbach’s Alpha calculated on the current sample was adequate (α = 0.74).

*Intolerance of Uncertainty Scale short form* (IUS-12) [100], Italian version [101]. It is a 12-item self-report measure that assesses one’s ability to cope with unpredictable changes and respond appropriately in inherently ambiguous situations. The items are rated on a five-point Likert scale (ranging from* 1* = *not at all characteristic of me* to* 5* = *entirely characteristic of me*). A total score was computed. In the present study, the overall internal consistency for the scale was good (α = 0.87).

*Acceptance and Action Questionnaire-II* (AAQ-II) [102], Italian version [103]. The AAQ-II is a measure of psychological inflexibility and experiential avoidance which are related to a wide range of psychological disorders and quality of life. In this study, the ten-item Italian version is evaluated on a seven-point Likert scale (*from 1* = never true to 7 = *always true*). It is a unidimensional measure where higher scores indicate greater psychological inflexibility. Cronbach’s Alpha in this study was good (α = 0.83).

*Connor Davidson Resilience Scale 10©* (CD-RISC 10) [104, 105], Italian version [106]. The CD-RISC 10 is a brief measure of resilience that examines one’s ability to cope with adversity. It consists of ten items are rated on a scale from 0 to 4 (*0* = not true at all to 4 = *true nearly all the time*). A higher score indicates greater resilience. In the current sample, the scale demonstrated good reliability (α = 0.85).

*Depression Anxiety Stress Scales-21* (DASS-21) [107], Italian version [108]. It is a self-report questionnaire with 21 items measuring depression, stress, and anxiety (seven items for each subscale) based on a four-point rating scale (with endpoints labelled *0* = *did not apply to me at all* and *3* = *applied to me much, or most of the time*). Higher scores indicate higher levels of depression, anxiety, or stress. In the current sample, Cronbach’s Alpha for Stress and Depression subscales were excellent (α = 0.90), and good for the Anxiety subscale (α = 0.84).

*Well-Being Numerical Rating Scales* (WB-NRSs) [109]. It is a five-item instrument that assesses physical, psychological, relational, spiritual, and general well-being. Each of the five items uses a 10-point numerical rating scale (with* 1* indicating a state of “*absolute distress*” and* 10* a state of *complete well-being*). The respondent selects an integer that best reflects the magnitude of the characteristic being investigated. Single ratings can be used to assess each specific component of well-being.

### Statistical analysis

Before conducting the analyses, the missing values in the data were examined. Listwise deletion was used when one or more answers to the I-PVDQ were missing. For the other scales, listwise deletion was used when a case had more than 10% of missing answers [110]. Otherwise, the case item mean was used to replace the missing value.

In the preliminary phase of the statistical analyses, item descriptives were computed to examine the distribution of responses across the items. Specifically, mean, standard deviation, range, skewness, and kurtosis values were examined to test the variability in the item responses and departures from normal distributions. Values outside the range of − 1 and 1 were considered indicators of non-normal distributions [111].

To test structural/internal validity, the factor structure of the I-PVDQ was assessed. The data file was randomly split into two parts to perform Exploratory factor analysis (EFA; *N* = 256) and Confirmatory factor analysis (CFA; *N* = 245). EFA was performed on FACTOR [112]–Version 12.1. Optimal implementation of Parallel Analysis (PA) [113] was conducted to identify the number of recommended factors and the Robust Unweighted Least Squares (RULS) estimation method was used with the Robust Promin rotation. CFA model testing was based on EFA results conducted using JASP 0.16.3 [114]. The diagonal weighted least squares (DWLS) estimation method was employed because the data measured with Likert-type ratings are ordinal in nature.

Chi-square test, Comparative Fit Index (CFI), Tucker–Lewis index (TLI), and Root Mean Square Error of Approximation (RMSEA) were used to evaluate the goodness-of-fit. Specifically, an RMSEA value of approximately 0.08 and 0.05 would suggest moderate and excellent model fit, respectively, and CFI and TLI in the range of 0.90–0.95 would suggest moderate and excellent model fit, respectively [36, 110].

Multigroup CFA was used to evaluate whether the scale was invariant across genders (male sample: *N* = 205, and female samples: *N* = 295). Specifically, a hierarchically nested series of CFA were applied. An unconstrained model was used as a baseline to test configural invariance (i.e., the two groups share the same factor structure). Then, three more restrictive models were tested, which include: a model in which factor loadings were constrained to be equal across groups (Metric), a model in which factor loadings plus intercepts were constrained to be equal across groups (Scalar), and a model in which factor loadings, intercepts plus error variances were constrained to be equal across groups (Strict). Models were compared using the chi-square-based likelihood ratio and the equality constraints were tested using the Comparative Fit Index difference (*ΔCFI*), and Root Mean Square Error of Approximation (*ΔRMSEA*). A *ΔCFI* value ≤ 0.01 supplemented by a change ≤ 0.015 in *RMSEA* would indicate invariance [116, 117].

Reliability was measured as the internal consistency of the PI and GA subscales using Cronbach's alpha (for comparison with previous studies) and McDonald’s omega (that must be preferred for short scales) [118] with a relative 95% confidence.

Bayesian statistical analyses were used to evaluate the relationships among I-PVDQ factors (PI and GA) and the variables in the study, and gender differences in GA and PI. Jeffreys’ Bayes Factor described the observed data using a priori and posterior distribution [120], which allowed quantification of evidence in favor of the alternative and null hypothesis [121]. Bayes Factors for evidence of alternative hypotheses is presented as an easy-to-interpret odds ratio that represents the magnitude of the difference: 1–3 as weak, 3–10 as substantial, 10–30 as strong, 30–100 as very strong, and > 100 as decisive [121]. All the Bayesian tests were performed using JASP 0.16.3. Specifically, Bayesian correlation tests and Bayesian independent sample *t*-tests were used to investigate construct and criterion validity. Positive low to medium correlations (0.20 < *r* < 0.45) between the I-PVDQ factors and fear of COVID-19, intolerance to uncertainty, psychological inflexibility, stress, anxiety, and depression measures were expected, while negative low to medium correlations were expected with resiliency and well-being measures. Finally, it was hypothesized that women showed higher levels of GA and PI. Therefore, a one-tail hypothesis was tested (i.e., the women’s scores are higher when compared to men’s scores).

## Results

After examining the missing values, five cases (1.0%) were deleted because of missing responses in the I-PVDQ scale, three cases (0.6%) were deleted since there were more than 10% of missing values and the other nine single case item entries were replaced by the mean value for the respective item.

### Descriptives

Item descriptive statistics are presented in Table 1. All response options were selected, and item means ranged from 2.40 (*SD* = 1.40) to 5.98 (*SD* = 1.60). Values of skewness and kurtosis were above the −1/+1 range for 8 items.

**Table 1:** Descriptives and factor loadings of the items of the Italian Perceived Vulnerability to Disease Questionnaire (I-PVDQ)

Item	Descriptives (*N* = 501)					Factor loadings			
	Range	*M*	*SD*	*Sk*	*Ku*	*EFA* (*N* = 256)		*CFA* (*N* = 245)	
						GA	PI	GA	PI
1	1‒7	5.98	1.60	− 1.69	1.95	0.50	–	0.52	–
2	1‒7	2.70	1.42	0.76	0.12	–	0.58	–	0.68
3	1‒7	4.53	2.06	− 0.28	− 1.33	0.62	–	0.42	–
4	1‒7	4.54	2.12	− 0.28	− 1.37	0.48	–	0.57	–
5	1‒7	4.33	1.70	− 0.08	− 1.01	–	0.24	––	0.23
6	1‒7	2.42	1.40	1.09	0.77	–	0.92	–	0.78
7	1‒7	3.94	2.03	0.16	− 1.33	0.86	–	0.81	–
8	1‒7	2.82	1.67	0.78	− 0.34	–	0.87	–	0.80
9	1‒7	3.51	2.13	0.46	− 1.20	0.36	–	0.49	–
10	1‒7	2.40	1.40	1.06	0.71	–	0.81	––	0.70
11	1‒7	5.11	1.87	− 0.77	− 0.66	0.46	–	0.55	–
12	1‒7	4.09	1.70	0.01	− 0.97	–	0.38	–	0.48
13	1‒7	4.57	1.80	− 0.49	− 0.82	0.45	–	0.39	–
14	1‒7	4.08	1.51	− 0.06	− 0.77	–	0.44	–	0.44
15	1‒7	2.93	1.87	0.83	− 0.46	0.73	–	0.66	–

*Sk* skewness, *Ku* kurtosis, *EFA* exploratory factor analysis, *CFA* confirmatory factor analysis. *GA* Germ Aversion, *PI* perceived infectability

### Factorial structure and reliability of the I-PVDQ (single group analyses)

*Exploratory factor analysis (EFA)* The Kaiser–Meyer–Olkin (*KMO*) measure of sampling adequacy indicated that the strength of the relationships between the 15 items was fair (KMO = 0.84). Bartlett’s test of sphericity, which tests the overall significance of all the correlations within the correlation matrix, was significant (Bartlett’s χ^2^(*N* = 256, *df* = 105) = 1566.7, *p* < 0.001), and indicated acceptability to proceed with the analysis. Parallel analysis results indicated that two factors should be retained based on the 95-percentile of random eigenvalues. The two-factor model accounted for 47% of the variance and all items loaded on the appropriate dimension (EFA factor loadings are presented in Table 1). Factor PI and Factor GA accounted for 32% and 15% of the variance in the data, respectively. Exploratory factor analysis supported the two-factor structure of the I-PVDQ, and factor loadings attested that each factor included the items that are deemed to measure perceived infectability (PI) and Germ Aversion (GA). The correlation between the two factors’ scores was low (0.15).

**Fig. 1:**
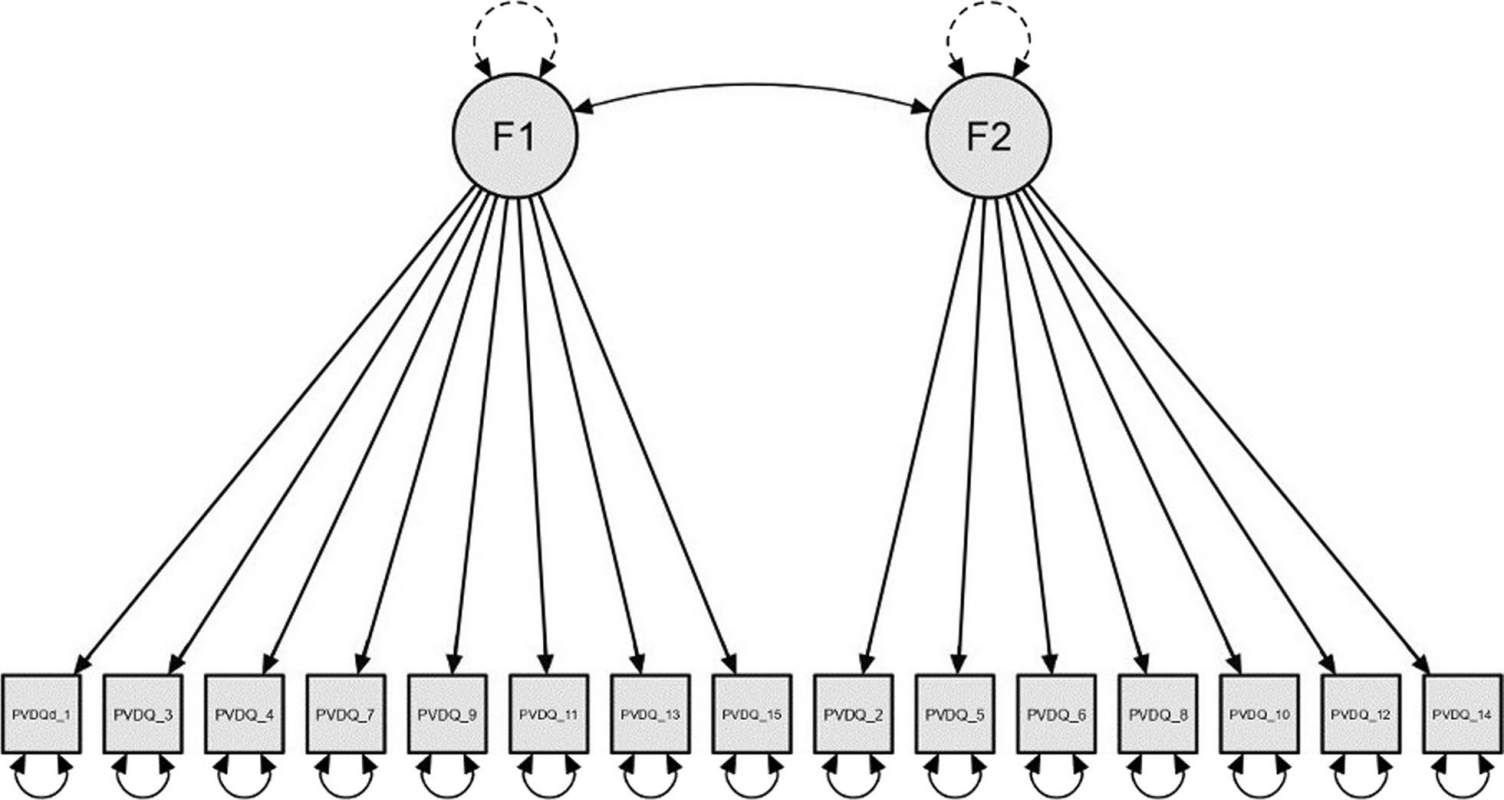
The two-factor model (F1 = Germ Aversion, F2 = perceived infectability) of the Italian Perceived Vulnerability to Disease Questionnaire (I-PVDQ)

*Confirmatory factor analysis (CFA)* By and large, CFA replicated the EFA results. Specifically, the fit of the two-factor model (Fig. 1) was good (*χ*^*2*^ (89) = 149.96, *p* < 0.001; *TLI* = 0.95; *CFI* = 0.94; *RMSEA* = 0.053 [90% CI: 0.038–0.067]). Factor loadings (Table 1) were all significant at *p* < 0.001 and ranged from 0.23 to 0.81. Factor covariance was equal to 0.16, indicating that the PI and GA were weakly correlated.

*Internal consistency* The internal consistency of the I-PVDQ was adequate for the PI (*α* = 0.76 [95%: 0.71–0.80], ω = 0.77 [95%: 0.72-0.81]), but slightly low for the GA (*α* = 0.67 [95%: 0.60–0.72], ω = 0.69 [95%: 0.63–0.75]).

### Invariance of the I-PVDQ across genders (multi-group analysis)

All the models showed an adequate fit and the difference in CFI and RMSEA values were all < 0.01 (Table 2). Thus, the scale maintains the equivalence of the two-factor structure (*Configural* invariance), factor loadings (*Metric* invariance), intercepts (*Scalar* invariance), and error variances (*Strict* invariance) across male and female groups.

**Table 2:** Fit Statistics of the Italian Perceived Vulnerability to Disease Questionnaire (I-PVDQ) invariance models across genders

Model	χ^2^ (*df*)	CFI	RMSEA	Model comparison	Δχ^2^ (Δ*df)*	*p*	ΔCFI	ΔRMSEA
*Gender*								
Configural	394.318 (178)	.927	.070	*–*	–	–	–	–
Metric	410.292 (191)	.926	.068	Metric-configural	15.974 (13)	< .025	− .001	− .002
Scalar	433.065 (204)	.923	.067	Scalar–metric	22.773 (13)	.05	− .003	− .001
Strict	468.308 (219)	.916	.068	Strict-scalar	35.243 (15)	< .005	− .007	.001

*df* degrees of freedom; CFI comparative fit index; RMSE Root Mean Square Error of Approximation; Δ difference between nested models; *p* probability value of Δχ^2^ test. *Metric* equality of factor loadings; *Scalar* = *Metric* + equality of intercepts, Strict Scalar + equality of error variances

### Correlations with related constructs and gender differences

*Perceived Infectability (PI)*. There is decisive evidence in favour of correlations between PI and other related constructs. A medium positive correlation was found with the measure of COVID-19-related fears (*r* = 0.40, BF_10_ > 100). Intolerance of uncertainty, psychological inflexibility, and resiliency were weakly related to PI (*r* = 0.21, BF_10_ > 100; *r* = 0.26, BF_10_ > 100; and *r* = − 0.24, BF_10_ > 100 respectively). Similarly, Stress, Anxiety, and Depression scores had low positive correlations with PI (*r* = 0.24, BF_10_ > 100; *r* = 0.22, BF_10_ > 100; and *r* = 0.24, BF_10_ > 100 respectively). Overall, correlations with wellbeing ranged from − 0.17 to − 0.21 with BF10 > 100 indicating negative low relationships with PI.

*Germ Aversion (GA)* There was decisive evidence of a medium positive correlation with the measure of COVID-19 related fears during the pandemic (*r* = 0.42, BF_10_ > 100). The evidence of the relationship was very strong between GA and IUS-12 (*r* = 0.16, BF_10_ > 30), and strong between GA and measure of Stress (*r* = 0.14, BF_10_ > 10). Bayesian correlations between GA and AAQ-II (*r* = 0.14), CD-RISC (*r* = − 0.10), and Anxiety (*r* = 0.10) were non-significant (BF_10_ < 10) while there was no correlation with the measure of Depression (*r* = 0.05, BF_10_ < 10). As for the well-being measures, decisive weak negative correlations have been observed GA and General well-being (r = − 0.18, BF_10_ > 100), and Relational well-being (r = − 0.17, BF_10_ > 100). Furthermore, there was evidence in favor of a strong negative relationship between GA and Physical well-being (r = − 0.14, BF_10_ > 10), and Psychological well-being (r = − 0.15, BF_10_ > 10). Finaly, there was not a correlation with Spiritual well-being (r = − 0.10, BF_10_ < 10). All correlations are reported in Table 3.

**Table 3:** Correlations between I-PVDQ subscales and all the other measures in the study

	I-PVDQ	
	Perceived infectability	Germ aversion
Fear of COVID-19	.40^***^	.42^***^
Intolerance to uncertainty	.21^***^	.16^**^
Psychological inflexibility	.26^***^	.13#
Stress	.24^***^	.14^*^
Anxiety	.22^***^	.10#
Depression	.24^***^	.05#
Resiliency	− .24^***^	− .10#
Physical well-being	− .21^***^	− .14^*^
Psychological well-being	− .21^***^	− .15^*^
Relational well-being	− .19^***^	− .17^***^
Spiritual well-being	− .17^***^	− .10#
General well-being	− .21^***^	− .18^***^

*N* = 501. I-PVDQ = Italian Perceived Vulnerability to Disease Questionnaire

#ns, *BF_10_ > 10, **BF_10_ > 30, ***BF_10_ > 100

*Gender differences* The one-sided Bayesian *t*-test showed a BF_10_ value of 0.44 for the PI factor, pointing out evidence in favor of the null hypothesis., i.e., perceived infectability was equal in men and women (*M* = 22.66, *SD* = 7.28, and *M* = 23.57, *SD* = 7.45, respectively). The result obtained for the GA factor (BF_10_ = 189.78) suggests substantial evidence in favor of the alternative hypothesis. The Germ Aversion score was substantially higher in women compared to men (*M*_*men*_ = 33.19, *SD* = 9.30 and *M*_*women*_ = 36.57, *SD* = 9.98, respectively).

## Discussion

The current study aims to investigate the psychometric properties of the I-PVDQ and considers the emotional, cognitive, and behavioral impact that the COVID-19 pandemic had on an individual’s perception of their vulnerability to illness. The investigation also provides evidence of the adequate psychometric properties of the Italian version of the scale which, to the best of our knowledge, has not been formally translated and validated.

Specifically, this study tested the I-PVDQ’s factorial structure and the gender invariance of the Italian version of the scale. In line with Duncan et al. [7], the exploratory factor analysis supported the two-factor structure of the I-PVDQ, and factor loadings attested that each factor included all the items that are deemed to measure Perceived Infectability (PI) and Germ Aversion (GA). Confirmatory factor analysis replicated these results. However, from both EFA and CFA, a low factor loading for PI item 5 was observed (“*My past experiences make me believe I am not likely to get sick even when my friends are sick*”). In fact, this item showed low factor loadings and was removed in other validation studies [25, 26], with an explanation that its reverse wording makes it harder to comprehend. The other possible explanation is that COVID-19 confronted people with a new and unknown, potentially fatal disease and beliefs about their vulnerability were challenged because they did not have “past” experience as a reference point. It is possible to consider that the pattern of responses to this item was influenced by the time of data collection when the main concern of the population was to protect themselves from contracting COVID-19, a highly infectious disease. As such, no one could consider themselves immune or rely on their previous experiences. A similar pattern of responses was evidenced by Do Bú and colleagues [24] who suggested that responses to the questions could be influenced by the prominent perceived contagiousness if the data collection period is during the strong prevention measures imposed by the government. Additionally, the relationship between beliefs about own vulnerability to illness and the discomfort due to potential pathogen transmission seems to be very weak during a pandemic. The prevention measures imposed to limit the spread of the disease (e.g., wearing masks, physical distancing, washing hands) had impacted people’s everyday spontaneous behavioural responses. Thus, the interpretation of the I-PVDQ items referring to GA changed, because some behaviours have been strongly discouraged (e.g., shaking hands) or recognized as highly dangerous (e.g., sharing the same drink bottle), and people may pay more attention to all those behaviours that usually were of no concern, such as handling money or sharing phones, regardless of how they judge their infectability.

Nonetheless, despite the low factor loading of item 5, this study provides evidence of the structural validity of the I-PVDQ replicating the original bi-factorial structure of the PVDQ [7] and maintaining all of the items. This result was not obtained in other studies that had to eliminate some items from the scale to reproduce the two factors and proposed shorter versions [24–26, 35].

As for internal consistency, the low indices for the GA subscale found in this study are in line with the pre- and post-pandemic literature [25, 29, 31, 34] and it could be explained by the heterogeneity of its questions. As noted by Diaz and colleagues [25], the GA subscale investigates a wide range of different behaviors (e.g., touching money, sharing a mobile phone, shaking hands), and the this characteristic may affect the internal consistency of the subscale [122].

The pattern of correlations of the I-PVDQ with pandemic-related constructs offered strong support for the existence of the expected relationships and provide evidence of the construct and criterion validity of the scale. The measure of COVID-19 related fears and both I-PVDQ factors (PI and GA) are linked, and the strength of the correlation was very similar to those reported by Ahorsu et al. [67], which employed the scale during the pandemic. As expected, we found evidence of the positive relationship between PI and intolerance to uncertainty, psychological inflexibility, stress, anxiety, and depression, as it was for the negative associations between PI, resiliency, and well-being. Although all these correlations were low, they suggest that perceived infectability is directly related to individual dispositions that impede coping with stressful situations, such as psychological inflexibility and intolerance to uncertainty, and inversely related to personal resources that help facing difficulties, such as resiliency. Consistently, all the aforementioned constructs related to PI have been identified as risk factors related to distress regarding the pandemic [47, 50, 52]. Moreover, the lack of previous experience and reference points, such as reliable information on transmission, pathogenicity, treatment, and prognosis of the new disease, probably affected PI and linked it to intolerance to uncertainty that is considered a major vulnerability factor involved in the development of several psychological disorders [80]. At the same time, we can suppose that PI contributed to the increase stress, depression, and anxiety [22, 61, 81–83]. The same pattern of correlations for GA subscale was not observed for depression, anxiety, psychological inflexibility, resiliency, and spiritual wellbeing, but we observed low correlations in the expected direction with stress and intolerance to uncertainty, (positive), and well-being (negative). A tentative explanation is that the attentive focus on the imposed rules to limit the spread of the disease, the lockdown restrictions, and the news on the pandemic statistics (e.g., infected and deceased people daily reports) impacted people’s behavioural responses to avoid germs, regardless their personal resources and level of distress.

Taken together, these results suggest that the I-PVDQ maintains its psychometric properties once translated and that when PVD becomes pervasive as it happens during a pandemic can be used to capture PVD changes in response to situations that are perceived as threatening to one’s health (e.g., [38, 39]). On one hand, perceived infectability refers to personal infection history and the belief that one is likely or unlikely to fall ill “outside” the pandemic context. Thus, people may lose their reference points “inside” a pandemic. Additionally, when the risk of infection is extremely high and dangerous (i.e., during the pandemic), PI appears linked to some psychological dispositions and well-being/distress indicators. On the other hand, GA is more related to behavioral responses. When all persons could be potential carriers of the virus and, consequently, everyone is asked to abstain from close social contacts and adopt hygiene practices, individual avoidance of potential infection sources inevitably changes [125–127] and appears to be unrelated (or very weakly associated) to psychological dispositions and distress indicators.

The impact of the pandemic on PVD is confirmed by gender differences observed in the current study. Contrary to Diaz et al. [25] a difference in GA, but not in PI was found. These findings could be a result of cultural differences or the fact that the broad perception of infectiousness during the pandemic overcomes gender distinctions. Previous findings noted gender differences in GA given higher pathogen disgust sensitivity in women (e.g., [39, 128]). Therefore, women can reinforce protective behavior by adopting appropriate hygiene practices and by avoiding situations that are associated with an increased risk of contracting the infection, such as close contact or physical proximity with other persons.

## Limitations

The present study is not without its limitations. One of the main limitations was the lack of pre-pandemic data to compare with those collected during the pandemic [129]. Furthermore, measures of other relevant variables (e.g., measures of fear of contamination, hypochondria, obsessive–compulsive symptoms, health status information) that might disentangle the nature of the PVD construct were not collected. Because of the particular historical moment (COVID-19 pandemic), a further limitation of this study is its local nature, as the sample consisted mainly of people residing in areas of Central Italy, as well as the lack of clinical sample. Indeed, whereas psychometric validation studies of PVDQ mostly availed of convenience samples, this aspect limits the generalizability of the results and did not allow certain aspects of PVD to be investigated, such as the relationship between age and the perceived infectability and germ aversion. Finally, future studies should aim to revise items to enhance the psychometric properties of the I-PVDQ and to highlight potential cultural differences in reactions to the pandemic. For example, item 4 includes content referring to using a pencil someone has chewed, item 15 refers to using public telephones, and item 11 refers to touching money. Given that the original measure was constructed in the early 2000s, some items may be less representative of people's everyday lives today (i.e., most people have their cell phones, many restaurants and shops encourage credit card payments, a lot of people write on their PC’s instead of using a paper and pencil). Furthermore, future research should consider revising the items so that they better reflect risk-reducing behaviors that have become part of our current way of behaving.

## Conclusions

Although environmental circumstances have changed substantively since the COVID-19 pandemic, the present study revealed that the I-PVDQ maintained its reliability and validity in an Italian sample albeit with a low factor loading in a single item. The Italian version of the PVDQ can be used to evaluate vulnerability to disease in the Italian-speaking sample, whereas future studies should explore the revision of specific items to reflect more accurately the current post-pandemic environment.

## Data Availability

The datasets used and/or analyzed during the current study are available from the corresponding author upon reasonable request.

## Abbreviations

AAQ-II: Acceptance and Action Questionnaire-II
CD-RISC 10: Connor Davidson Resilience Scale 10©
CFA: Confirmatory factor analysis
CFI: Comparative Fit Index
DASS-21: Depression Anxiety Stress Scales-21
DWLS: Diagonal weighted least squares estimation method
EFA: Exploratory factor analysis
GA: Germ Aversion
IUS-12: Intolerance of Uncertainty Scale short form
KMO: Kaiser–Meyer–Olkin measure of sampling adequacy
M: Mean
MAC-RF: Multidimensional Assessment of COVID-19-Related Fears
PA: Parallel Analysis
PI: Perceived Infectability
PVD: Perceived Vulnerability to Disease
PVDQ: Perceived Vulnerability to Disease Questionnaire
I-PVDQ: Italian Perceived Vulnerability to Disease Questionnaire
RMSEA: Root Mean Square Error of Approximation
RULS: Robust Unweighted Least Squares
SD: Standard deviation
TLI: Tucker–Lewis index
WB-NRSs: Well-Being Numerical Rating Scales
ΔCFI: Comparative Fit Index difference
ΔRMSEA: Root Mean Square Error of Approximation
